# Assessment in Undergraduate Competency-Based Medical Education: A Systematic Review

**DOI:** 10.7759/cureus.58073

**Published:** 2024-04-11

**Authors:** Sandeep K Gupta, Tripti Srivastava

**Affiliations:** 1 Pharmacology, Heritage Institute of Medical Sciences, Varanasi, IND; 2 Physiology, Datta Meghe Institute of Medical Sciences, Wardha, IND

**Keywords:** summative assessment, formative assessment, workplace based assessment, undergraduate medical education, competency based medical education, assessment

## Abstract

Background: Studies that have methodically compiled the body of research on the competency-based medical education (CBME) assessment procedure and pinpointed knowledge gaps about the structure of the assessment process are few. Thus, the goals of the study were to create a model assessment framework for competency-based medical education that would be applicable in the Indian setting as well as to thoroughly examine the competency-based medical education assessment framework. Methods: PubMed, MEDLINE (Ovid), EMBASE (Ovid), Scopus, Web of Science, and Google Scholar were the databases that were searched. The search parameters were restricted to English language publications about competency-based education and assessment methods, which were published between January 2006 and December 2020. A descriptive overview of the included research (in tabular form) served as the foundation for the data synthesis. Results: Databases provided 732 records; out of which 36 fulfilled the inclusion and exclusion criteria. Thirty-six studies comprised a mix of randomized controlled trials, focus group interviews, and questionnaire studies, including cross-sectional studies, qualitative studies (03), mixed-method studies, etc. The papers were published in 10 different journals. The greatest number was published in BMC Medical Education (18). The average quality score for included studies was 62.53% (range: 35.71-83.33%). Most authors are from the UK (07), followed by the USA (05). The included studies were grouped into seven categories based on their dominant focus: moving away from a behavioristic approach to a constructive approach of assessment (01 studies), formative assessment (FA) and feedback (10 studies), the hurdles in the implementation of feedback (04 studies), utilization of computer or online based formative test with automated feedback (05 studies), video feedback (02 studies), e-learning platforms for formative assessment (04 studies), studies related to workplace-based assessment (WBA)/mini-clinical evaluation exercise (mini-CEX)/direct observation of procedural skills (DOPS) (10 studies). Conclusions: Various constructivist techniques, such as concept maps, portfolios, and rubrics, can be used for assessments. Self-regulated learning, peer feedback, online formative assessment, an online computer-based formative test with automated feedback, the use of a computerized web-based objective structured clinical examination (OSCE) evaluation system, and the use of narrative feedback instead of numerical scores in mini-CEX are all ways to increase student involvement in the design and implementation of the formative assessment.

## Introduction and background

The goal associated with medical courses is to effectively prepare future physicians to meet the healthcare needs of the general public. The evaluation methods in India's previous medical education curriculum placed a greater emphasis on knowledge than on competencies. Summative evaluation had a higher degree of weight than formative evaluation with feedback. As a result, doctors trained under prior curricula had strong theoretical understanding but lacked practical experience and other soft skills like professionalism, ethics, and communication [1]. The implementation of competency-based medical education (CBME) was primarily intended to address the aforementioned deficiencies [1,2].

The CBME framework places a strong emphasis on the competencies required to meet the needs of patients. This method places a strong emphasis on connecting teaching, learning, and assessment to actual medical practice. Effective CBME assessment is characterized by certain essential elements. It must be regular and ongoing. This will enable more formative assessments to be conducted to direct the students' development. The majority of the assessment must be work-based. A crucial element of CBME would be the direct observation and evaluation of real-world clinical interactions. The assessment instruments themselves have to adhere to a set of minimal requirements for quality in terms of reliability, validity, affordability, educational impact, and dependability [1,2].

In competency-based medical education, assessments are not meant to serve as a final judgment, but rather to help students advance to the next level of expertise. Even though written exams are typically employed to assess students, assessments that primarily rely on direct observation to measure skill performance offer more convincing proof that learning objectives have been met. Effective feedback is essential for supporting a learner's professional growth once evaluations have identified their strengths and areas for improvement. Formative assessments are given increasing importance, and feedback is a crucial component of them. Additionally, each student's proficiency is evaluated using objective, quantifiable criteria [3].

Competencies are discernible, quantifiable capabilities that educators want their students to acquire. Knowledge, skills, attitudes, and communication are outlined as areas of competency [3]. In contrast to conventional education, which places a strong emphasis on subject matter, CBME doesn't start by considering the appropriate amount of content to teach. The main objective of CBME is to measure the outcomes of student performance that students must achieve to show their competency. As a result, the focus changes from assessing input to assessing output or outcomes [4].

A medical student must be evaluated for us to authorize them to handle human life. In the end, the healthcare educational community has a professional duty to the general population to guarantee that its students are capable enough to practice independently. Within the framework of CBME, assessment refers to the procedure that allows for the testing of knowledge, skills, and attitudes to determine competency [5].

The foundation of any curriculum is assessment. All medical educators would concur that assessments are still the most effective way to promote learning and that an assessment that is not connected with the learning objectives cannot accomplish its goals [6]. However, a major weakness in the CBME is the lack of reliable and accurate assessment methods. Appropriate assessment techniques are crucial to the effective implementation of CBME; yet there is still a lack of clarity around the assessment instruments, process settings, and modalities and timeframes of CBME assessments [2,7].

The finest available evidence must serve as the foundation for the CBME evaluation framework's planning. We must use the most effective evaluation techniques to satisfy CBME's requirements. Multiple assessment instruments with low validity risks should be included. To make the CBME assessment plan more robust, many additional objective-type settings are required. It is also necessary to include qualitative as well as quantitative evaluation techniques, especially when evaluating non-cognitive skills [8,9].

We need to add more assessment tools to our toolkit for CBME. Given the abundance of evaluation possibilities, these tools ought to be simple to use, enable speedier decision-making for corrective action, be workable, and possess sufficient rigor to be deemed appropriate [10-14].

To solve several significant issues concerning the CBME assessment scheme, more data is required. However, there aren't many studies that have methodically compiled the body of research on the CBME assessments and pinpointed information gaps about the assessment process's framework. This study aims to fill that gap. Hence, one of the primary objectives of this study was a systematic review of the competency-based medical education assessment framework with reference to the assessment instruments, process settings, and modalities. Building a competency-based medical education model assessment system that may find application in the Indian setting was another objective of this study.

## Review

Methods

Information Sources

PubMed, EMBASE, Scopus, Web of Science, and Google Scholar were the databases that were searched.

Search Strategy

The search parameters were restricted to English-language publications pertaining to competency-based education and assessment methods that were published between January 2006 and December 2020. We incorporated the relevant database-specific restricted vocabulary words and keyword combinations for every topic in our search approach. Boolean operators were then used to combine these terms, which were then used for database searches. Competency-based medical education (CBME), competency, competence, clinical competence, clinical skills, assessment, assessor, assessment tools, feedback, criterion-referenced evaluation, simulation, objective structured clinical examination (OSCE), workplace-based assessment (WBA), mini-clinical evaluation exercise (mini-CEX), portfolio, multi-source feedback (MSF), reflective practice, and directly observed procedural skills (DOPS) were the key terms used.

Eligibility Criteria

Studies were included if the research's setting was competency-based medical education (CBME); if the research focused on assessment tools or activities associated with CBME; if the research was empirical primary research; if the research was quantitative, qualitative, or mixed-methods; if research works were published between January 2000 and December 2020; and if the article was written in English.

Studies were excluded if they were systematic reviews, narrative reviews, reviews, commentaries, evidence-based perspectives, educational forums, commentaries, short communications, gazetted notifications, guidelines, case reports, abstracts, and editorials, and if they were not published in peer-reviewed journals.

Data Extraction

Rayyan, a software for systematic reviews, was used to upload each article. A free online tool called Rayyan helps writers with systematic reviews with their literature screening.

Following a preliminary filter for duplicates and search stipulations, the remaining papers' titles and abstracts were examined. The articles' entire texts were then evaluated by the inclusion and exclusion criteria to make the final selection.

A two-step screening procedure was used. The first author finished the initial step, which involved removing any duplication and reviewing titles before moving on to abstracts. To determine which research qualified for inclusion, the two authors thoroughly reviewed each study in relation to the criteria in the second stage. To reach a consensus, the disagreements were discussed. Any differences of opinion on the suitability of the research were settled by consensus.

The Preferred Reporting Items for Systematic Reviews and Meta-Analyses (PRISMA) guideline was used for describing the identification, screening, eligibility, and final inclusion of the papers. The purpose of research study quality evaluation instruments is to evaluate specific research designs. An appraisal of the quality of evidence is often used to assess the risk of bias. More recently, quality appraisal tools have been framed for appraising reviews with diverse designs. Because this review included studies with various designs, the Quality Assessment Tool for Studies with Diverse Designs (QATSDD) was used to evaluate the article's quality. This tool was published in 2012 to appraise the methodological quality, evidence quality, and quality of reporting in reviews that included studies with heterogenous designs (i.e., qualitative, quantitative, mixed- and multi-methods research) using a single tool. It contains 16 reporting criteria scored on a scale from 0 to 3. The QATSDD tool has a total of 16 criteria, of which 14 apply to qualitative studies, 14 apply to quantitative studies, and all 16 apply to mixed methods research. The QATSDD criteria assess the following: theoretical framework, aims/objectives, sample size, representative sample of the target group, the rationale for the choice of data collection tool, the rationale for the choice of data collection tool, detailed recruitment data, statistical assessment of the reliability and validity of the measurement tools, the fit between stated research question and method of data collection, the fit between stated research question and format and content of data collection tool, fit between research question and method of analysis, justification for the analytical method selected, assessment of the reliability of the analytical process, evidence of user involvement in design, strengths, and limitations critically discussed. The quality score of each included study was used to assess the risk of bias. As mentioned above, the QATSDD tool consists of 16 items (with some items only applicable to quantitative and qualitative studies). It is scored on a Likert scale from 0 = “high risk of bias” to 3 = “minimal risk of bias” and has strong reliability and validity in scoring studies with various (mixed) designs. A QATSDD overall percentage score was calculated for each included study. A study with a “low risk of bias” has an overall QATSDD percentage score greater than or equal to 75%. A study with a “moderate risk of bias” has an overall QATSDD percentage score between 50 and 74%, while a study with a “high risk of bias” has an overall QATSDD score between 0 and 49% [15].

Data Synthesis

A standard data extraction form was used for the data extraction process. The study authors, the year of publication, the corresponding author's country, the journal, the study type, the study setting, the study population or sample size, data collection, and analysis method, study outcome, and quality score were extracted from the studies according to a standard format. Textual narrative synthesis was used for data synthesis and analysis. This involves synthesizing the findings from primary studies textually. This method was adopted because it has proved useful in synthesizing evidence of different types (qualitative, quantitative, mixed method, etc.). This approach enabled this review to synthesize findings from both qualitative and quantitative studies to provide a comprehensive synthesis of the research literature in this field.

A descriptive overview of the included research presented in tabular form served as the basis for the synthesis. The key findings of each of the included studies were tabulated by the reviewers to answer the research question. This systematic review came to a qualitative conclusion by analyzing, contrasting, and summarizing the findings of the different investigations. All data types were given equal weight since the results of all the empirical studies were incorporated into a narrative form for this systematic review. Following the extraction of data, the studies were analyzed and classified as competency-based medical education methods of assessment. We undertook conceptual mapping to identify themes within which to synthesize and present the findings of primary studies. The results were presented in a structured manner by dividing the studies into the following various homogenous categories based on their dominant focus: The evidence gathered through this systematic review was graded using the Grading of Recommendations Assessment, Development, and Evaluation (GRADE) method [16].

Ethical Consideration

The Heritage Institute of Medical Sciences Varanasi's Institutional Ethics Committee (IEC) gave its approval for this study via letter no. HIMS/IEC/84/2022 dated March 7, 2022.

Result

**Figure 1:**
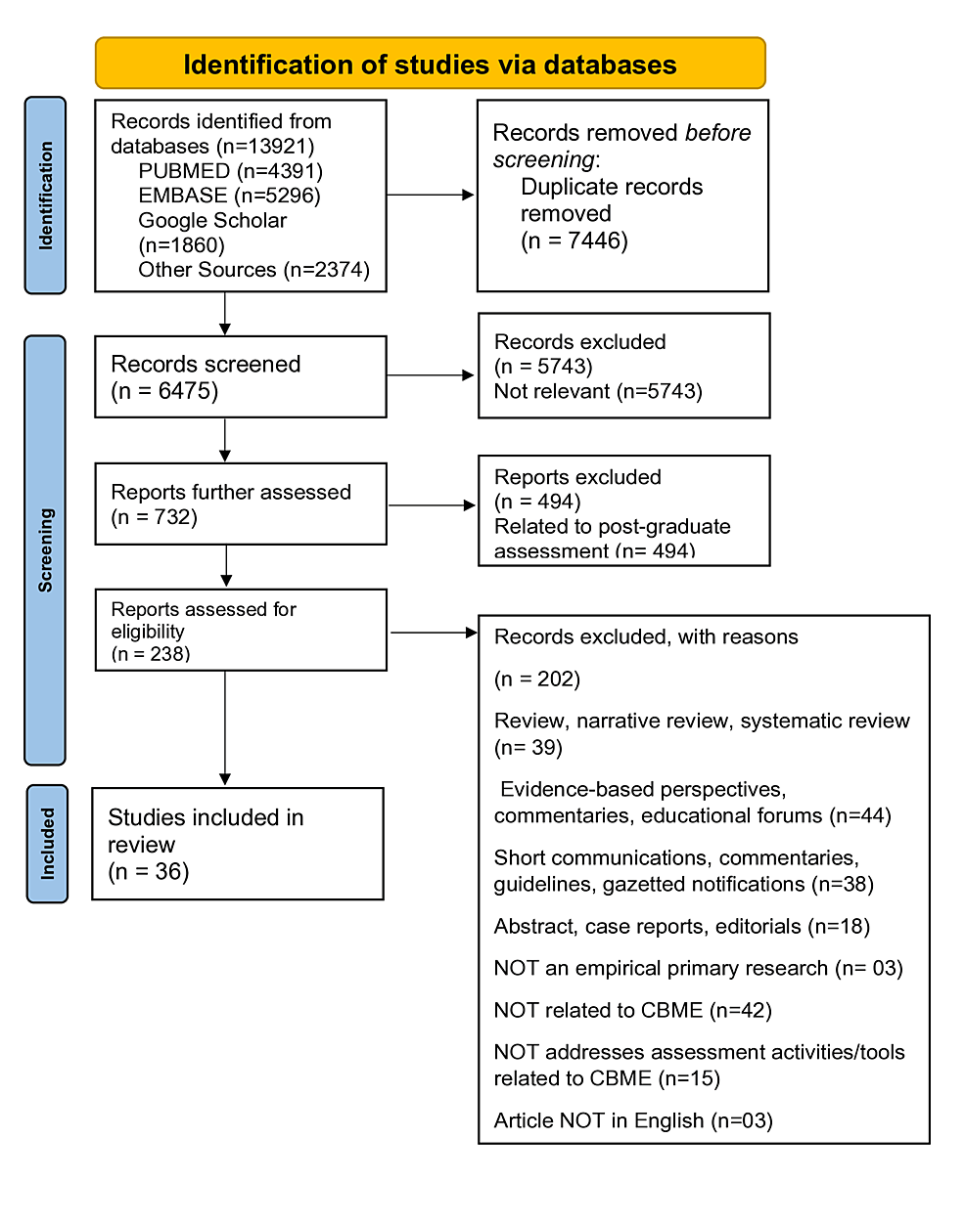
Flow diagram according to preferred reporting items for systematic reviews and meta-analyses (PRISMA)

Databases provided 13921 records [PUBMED (n=4391); EMBASE (n=5296); Google Scholar (n=1860) and Other Sources (n=2374)]; from this initial list, 7446 were removed given that they were duplicates. These 7446 records were further screened, and 5743 records were excluded as they were not relevant. 732 reports were further assessed, and 494 reports were excluded because they were related to post-graduate assessment. Thus, 238 reports were assessed for eligibility, and 202 were excluded according to the inclusion and exclusion criteria. Thus, a total of 36 studies remained eligible to be included for review (Figure 1). The identification, screening, eligibility, and final inclusion of the papers have been described using the PRISMA flowchart of systematic review (Figure 1).

Description of the Included Studies

The 36 studies comprised a mix of randomized controlled trials (02), prospective cohort studies (01), quasi-randomized trials/quasi-experimental studies (02), randomized crossover studies (01), controlled crossover trials (01), focus group interviews (02), questionnaire studies including cross-sectional studies (15), qualitative studies (02), qualitative studies with phenomenological designs (01), mixed-method studies (03), observational studies (02), multi-level analysis (02), feasibility studies (01), and principal component analysis (01). Table 1 lists the included studies along with their characteristics. The average quality score for included studies was 62.53% (range: 35.71-83.33%). Five studies had a “low risk of bias” with an overall QATSDD percentage score greater than or equal to 75%. Twenty-eight studies had a “moderate risk of bias” with an overall QATSDD percentage score between 50 and 74%, whereas three studies had a “high risk of bias” with an overall QATSDD score between 0 and 49% (Table 1).

**Table 1:** The included studies along with their characteristics

Author	Year	Country	Journal	Study Type	Study setting	Study Population/Sample size	Data Collection and analysis Method	Outcome	Quality score/ overall (%)
Harrison CJ [17]	2016	UK	Perspect Med Educ	Focus Group Discussion	Cleveland Clinic Lerner College of Medicine, USA, Keele University School of Medicine, UK, Maastricht University, Netherlands	Six focus groups were conducted in April to June 2014 (two at each school)	Constructivist grounded theory approach	Students should be enabled to have greater control over assessment and feedback processes.	64.28
Kishan Prasad HL et al. [18]	2020	India	Medical Science Educator	Cross-sectional Study	Department of Pathology, K S Hegde Medical Academy of Nitte, Mangaluru, India	One hundred four students participated in the study, but 89 students appeared for both sets of practical examination; hence, only these were included	Comparison of scores obtained by OSPE in comparison with conventional practical examination	OSPE as a formative assessment tool will help in modifying teaching-learning strategies	40
Lim YS [19]	2019	USA	Medical Science Educator	Questionnaire based study	Zucker School of Medicine, USA	A total of n = 140 students completed the questionnaire	Questionnaire study with Multivariate statistical method to analyze the data.	There is role of formative assessments to teach self-directed learning skills; but increasing student involvement is needed	64.28
Wolcott MD et al. [20]	2018	USA	BMC Medical Education	A principal component analysis (PCA)	University of North Carolina at Chapel Hill (UNC) Eshelman School of Pharmacy	All first-year students (n = 148) completed 5 capstone- multiple mini interview (C-MMI) stations.	Multifaceted Rasch Measurement (MFRM) model assessed student performance	The multiple mini-interview (MMI) can be a valuable assessment strategy	50
Hossain S [21]	2014	Bangladesh	Bangladesh Journal of Medical Education	Cross sectional descriptive study	Government and private medical Colleges, Dhaka city	79+27 (teachers from government and non-government medical colleges	Completed data and questionnaire	Formative assessment has got significant effect on summative assessment in various aspects.	54.76
McKenzie S [22]	2017	Australia	Advances in Medical Education and Practice	Qualitative and Quantitative study	Central Clinical School, Sydney Medical School, Australia	Two cohorts of interns, (total n=66) were invited to complete an anonymous survey.	Questionnaire results recorded as categorical variables. Free text responses collated and thematically analyzed	Procedural skills training, with feedback, should be universal.	58.33
Gonzalo JD [23]	2014	USA	BMC Medical Education	Multi-center qualitative study	10 academic US institutions	Internal medicine attending physicians (n= 34) identified as respected bedside teachers from 10 academic US institutions	Thematic analysis qualitative study using transcripts from audio-recorded, semi-structured telephone interviews	Clinical teachers should be encouraged to incorporate feedback and reflection strategies into their bedside teaching	71.42
Choi S et al. [24]	2020	Korea	BMC Medical Education	Randomised controlled trial	Seoul National University College of Medicine (SNUCM), Korea	87 medical students	A linear mixed effects model to compare the baseline and final test scores	Practicing written clinical cases with reflection and feedback is superior to a lecture-based approach	59.5
Kim EJ and Lee KR [25]	2019	South Korea	BMC Medical Education	A quasi-experimental posttest design	Hallym University, South Korea	110 participants randomized to a positive feedback (PF) group (n = 58) and a negative feedback (NF) group (n = 52).	After delivery of feedback, the participants assessed their own performance	Evaluator’s verbal feedback exerts a significant influence on the accuracy of self-assessment	59.52
Uhm S [26]	2015	UK	Medical Education Online	Cross-sectional study	Chung Ang University Hospital, South Korea	Final Medical students (n=87). Video-recordings of 26 students were randomly selected for qualitative analysis.	Communication skills scores before and after receiving feedback based on qualitative analysis.	Incorporating feedback for communication skills assessment gives essential information to learn and self-reflect	58.33
Pelgrim EAM et al. [27]	2012	Netherlands	Medical Education	Questionnaire study	Two institutions in the Netherlands	Of the 27 trainees who responded, authors selected a purposeful sample of 22 trainees	Semi-structured Interviews data analysed using a qualitative, phenomenological approach	The content of feedback, the way it is provided and its incorporation in trainees’ learning are important	78.57
Bok HGJ [28]	2013	Netherlands	BMC Medical Education	Questionnaire and group interviews	Faculty of Veterinary Medicine, Utrecht University (FVMU) in the Netherlands	85+18 students	Group Interviews (Quantitative +Qualitative data analysis)	Providing simultaneously formative feedback and input for summative decisions proved not easy to implement.	54.76
Bates J et al. [29]	2013	Canada	Medical Education	The methodological approach used was constructivist grounded theory (CGT)	Two schools of medicine in western Canada, the University of British Columbia (UBC) and the University of Alberta (UA).	19 students invited and 13 recruited	Individual semi-structured Interviews and an iterative coding process.	Assessment and feedback are constructive if embedded in daily patient care and are longitudinal	64.28
Harrison CJ [30]	2013	UK	Medical Education	Cross Sectional Study	Keele University School of Medicine	138 students completed a questionnaire	Latent class analyses	We need to construct feedback after summative assessment	69.04
Feller K and Berendonk C [31]	2020	Switzerland	BMC Medical Education	Qualitative study	Department of Diabetology at the University Hospital of Bern	Three focus group interviews: residents (six), with the supervising physicians (seven), and the allied health care professionals (eight).	Focus group discussions	Feedback from allied health care professionals can be a valuable learning resource for residents	61.90
Mitra NK [32]	2015	Malaysia	BMC Medical Education	Quasi-randomized trial	International Medical University, Kuala Lumpur, Malaysia	The control group (n = 102) ;The experimental group (n = 65)	Paper-based formative MCQ test; online formative MCQ tests with automated feedback	Computer based formative test with automated feedback improved the performance of students.	61.90
Bijol V [33]	2015	USA	Medical Education Online	Cross-sectional study	Harvard Medical School	Second-year medical and dental students (total n=161).	Those who used tool(‘quizzers’) and did not use the tool (‘non-quizzers’) was compared.	Students who chose to use quizzes did well on all aspects of the final exam	64.28
Palmer E and Devitt P [34]	2014	Australia	BMC Medical Education	A randomised controlled trial	University of Adelaide, Adelaide, Australia	The Year 1 (129 students). In Year 2 (130 students)	Outcomes from pre and post testing to evaluate assessment of a structured online formative assessment program.	The online medium is a valuable and appreciated resource, however, the production of quality content is a time-consuming exercise	57.14
Kühbeck F et al. [35]	2019	Austria	BMC Medical Education	A prospective cohort study	Technical University of Munich (TUM).	Cohort of 393 first-year medical students enrolled in a general pharmacology course	To collect quantitative data in real time, a web-based learning analytics platform was developed	Formative feedback by online assessments help students to better judge their academic performance.	76.19
Ode GE et al. [36]	2019	USA	Journal of Surgical Education	Feasibility study that prospectively assesses the implementation of a web-based O-SCORE	An academic medical center	The study included only residents in postgraduate training years (PGY) 2 to 5 (n = 20).	Data were compiled in the REDCap database and exported to Statistical Analysis System for analysis.	An immediate feedback program utilizing an electronic platform offers reproducible construct validity.	64.28
Kam BS et al. [37]	2019	South Korea	BMC Medical Education	A questionnaire-based	Pusan National University School of Medicine, South Korea	Two identical online surveys of Year 1 medical students (N = 103)	Students’ perceptions about clinical performance assessment (CPA) scores before and after video feedback.	Video feedback system might help students recognize their results	66.66
Dohms M.C. et al. [38]	2020	Brazil	BMC Medical Education	Pre/post study with a control group	Integrated primary care program in Brazil	First-year medical residents (N: 61)	Scores of the questionnaires pre- and post interventions	Video Feedback leads to deeper level of self-assessment, peer-feedback, and reflective practices.	77.08
Ismail MAA et al. [39]	2019	Malaysia	BMC Medical Education	Qualitative study with phenomenological design	USM School of Medical Sciences, Malaysia	Five focus groups discussion (FGDs) with medical students who had participated in several Kahoot! sessions	Qualitative methods.	The Kahoot platform is a promising tool for formative assessment in medical education.	66.66
Jafri L et al. [40]	2020	Pakistan	BMC Medical Education	Descriptive multimethod study	Department of Pathology and Laboratory Medicine, Aga Khan University (AKU), Karachi, Pakistan	Six Post Graduate Students and seventeen assessors	Annual assessment scores before and after implementation of WBA was assessed (Quantitative and Qualitative analysis)	Virtual learning environment (VLE) for execution of WBA program should be encouraged	68.75
Denison A [41]	2016	UK	Perspect Med Educ	Cross sectional study	School of Medicine, University of Aberdeen, Aberdeen, UK	A total of 558 and 498 examiner-candidate interactions in the January OSCE examinations, and 1402 and 1344 for the May OSCE examination for 2012 and 2013 respectively.	Examiner comments were analyzed for quantity and quality.	Use of tablet devices in OSCE assessments leads to improved examiner comment for use as feedback	59.52
Schmidt T et al. [42]	2020	Germany	BMC Medical Education	Controlled crossover study	Institute of Medical Genetics and Applied Genomics, University of Tuebingen, Germany	154 students of Human Medicine	Questionnaires and quantitative analysis of the data	Impact of Audience response systems on summative assessments may be limited	42.85
Nair BR [43]	2015	Australia	Advances in Medical Education and Practice	Focus groups, interviews, and surveys.	Centre for Medical Professional Development, Newcastle, NS W, Australia,	Five WBA cohorts (n=95) participate in one of two candidate focus groups. All assessors in the WBA program (n=72) attended assessor focus group	Focus group discussion and a grounded theory-based qualitative analysis	WBA process was positive and presented good opportunities for performance improvement	69.04
Lefroy J [44]	2015	UK	Medical Education	Randomised crossover study	Keele University School of Medicine	There were 144 students in the year cohort. Of these, 110 volunteered to participate.	A realist approach to understand the impact of feedback with and without grades	The use of grading in the provision of more effective, tailored feedback	66.66
Gaunt A [45]	2017	UK	Medical Education	Mixed-methods design	A mixture of large university hospitals and district general hospitals in several UK regions	10 focus group discussions with 42 surgical trainees	Focus group discussions with template analysis approach	Providing feedback during WBA is not emotionally neutral	75
Liang Y and Noble LM [46]	2020	China	MEDICAL EDUCATION ONLINE	Questionnaires based study	Guangzhou Medical University, Guangzhou, China	Online questionnaires (N = 91) and interviews (N = 22)	Online questionnaires and audio-recorded interviews	Participants didn’t fully understand the formative purpose of mini-CEX, particularly supervisors	50
Berendonk C et al. [47]	2018	Switzerland	BMC Medical Education	Multilevel factor analysis	4th-year medical students	A total of 1773 mini-CEX from 164 students were analyzed.	Students and clinical supervisors rated the students’ performance on a 10-point scale.	These findings put a question mark on the validity of mini-CEX domain scores for formative purposes	83.33
Rogausch A [48]	2015	Switzerland	BMC Medical Education	A multilevel analysis	Institute of Medical Education, University of Bern, Bern, Switzerland	A total of 512 trainers in 45 clinics provided 1783 mini-CEX ratings for 165 students	Linear regression analyses used to predict mini-CEX scores by OSCE performance and characteristics of clinics, trainers, students and assessments.	mini-CEX scores seem to have little informational value. Narrative feedback is more suitable.	73.80
Suhoyo Y et al. [49]	2020	Indonesia	BMC Medical Education	Questionnaire based study	Faculty of Medicine, Universitas Gadjah Mada, Indonesia	130 students	Questionnaire	Students and specialists were positive about the mini-CEX	54.76
Liao KC [50]	2013	Taiwan	BMC Medical Education	Observational, two-phase study	Department of Internal Medicine, Chang Gung Memorial Hospital, Chang Gung University, College of Medicine, Taiwan	863 clinical encounters of mini-CEX, which involved 97 residents and 139 evaluators	In the first phase, two-hour mini-CEX workshop. In the second phase, the data of monthly mini-CEX of internal medicine residents were collected and analyzed	Faculty development is a prerequisite to train evaluators in order to implement a successful mini-CEX assessment program.	64.28
Tokode OM and Dennick R [51]	2013	UK	International Journal of Medical Education	A qualitative study	University of Nottingham (two University affiliated district general hospitals)	60 foundation year-one trainees from the 2007/2008 cohorts of house officers in the two hospitals were invited for the study. Fifty (83.3%) trainees responded to the invitation to participate in the study.	Data were collected via semi-structured focus group Interviews. Data were analysed using the template thematic procedure.	There was theoretical formative potential of mini-CEX, but in reality educational experiences with it are mixed.	64.28
Bansal M [52]	2019	India	Indian J Otolaryngol Head Neck Surg	Cross-sectional study	E.N.T. Department of medical college hospital.	Thirty-three trainees and five trainers	Assessment for each DOPS encounters by structured standardized rating scale	DOPS is a high-quality instrument as it tests the candidate at the ‘‘does’’ level	35.71

Journals

The papers included in this systematic review were published in 10 different journals. The greatest number of articles were published in BMC Medical Education (18), followed by Medical Education (05), Medical Education Online (03), Medical Science Educator (02), Perspectives on Medical Education (02), Advances in Medical Education and Practice (02), Indian J Otolaryngol Head Neck Surg (01), Journal of Surgical Education (01), Bangladesh Journal of Medical Education (01) and International Journal of Medical Education (01).

Countries

For the geographical distribution analysis of articles, the country of origin of the first author was taken into consideration. The greatest number of authors were from the UK (07), followed by the USA (05), Australia (03), South Korea (03), Switzerland (03), the Netherlands (02), India (02), Malaysia (02), Bangladesh (01), Brazil (01), Canada (01), Taiwan (01), Pakistan (01), Germany (01), Indonesia (01), China (01), and Austria (01).

The included studies were grouped into the following seven categories based on their dominant focus (Table 2).

**Table 2:** The seven categories of included studies were based on their dominant focus and their important features

*Transitioning from a behavioristic assessment method to a constructive one*
(i) Need to abandon behavioristic assessment methods that rely on incentives and punishments. (ii) Recommendation of using three constructivist assessment tenets: step-by-step descaffolding to facilitate change towards a learning orientation, authenticity, and giving students a more active role.
Formative assessment and feedback
(i) Using the Objective Structured Practical Examination (OSPE) as a formative assessment tool. (ii) Multiple mini-interviews (MMI) can be a useful tool. (iii) Too many formative assessments interfere with students' ability to learn independently. (iv) Checklists, together with timely feedback, help learners recognize their mistakes. (v) Process-oriented feedback is advantageous over feedback that is outcome-oriented. (vi) Compared to negative feedback, positive feedback results in higher levels of self-efficacy and stronger positive feelings. (vii) Feedback-based communication skills assessments may result in better communication skills.
Hurdles in the implementation of feedback
(i) Time restrictions prevented high-quality feedback from being provided by supervisors. (ii) Students were reluctant to request assessments with feedback because they thought all workplace-based assessments (WBAs) were summative. (iii) Narrative formative input was deemed unhelpful by the trainees. (iv) Developing a clinical setting that is inherently supportive of feedback. (v) Providing supervisors and students with feedback training
Computer or online-based formative test with automated feedback
(i) Favourable correlation between how well one performed in the summative assessment and the online computer-based formative assessment with automated feedback. (ii) Online quizzes that provide relevant feedback, known as formative self-assessments, can assist students in evaluating their knowledge and pinpointing areas of weakness.
Video feedback
(i) Video feedback was more useful than the analytical checklist score. (ii) Real-world video feedback appears to be significantly linked to an increase in one's perception of one's own empathy.
e-learning platforms for formative assessments
(i) Potential for the Kahoot! platform to help with formative evaluation in medical education. (ii) Real-time data collection for formative assessments is possible with virtual learning environments (VLEs). (iii) The usage of tablet devices in OSCE exams is linked to better examiner comments. (iv) The primary benefits of audience response systems would be more student motivation and the creation of an engaging learning environment than an increase in assessment scores.
Workplace-based Assessment (WBA)/mini-clinical evaluation exercise (mini-CEX)/Direct Observation of Procedural Skills (DOPS)
(i) The degree of trainees' ambiguity over WBA's goal greatly influenced how they used it. Many trainees thought of WBA as a way to evaluate their learning, and some weren't sure what WBA was there for. (ii) Participants, in particular instructors, were not completely aware of the mini-CEX's formative aim. (iii) Low acceptance of mini-CEX by some learners may be caused by the high-pressure hospital setting with overlapping clinical responsibilities, the challenge of organizing the assessment, and the variable availability of time as well as the motivation of consultants to deliver quality feedback.

Transitioning From a Behavioristic Assessment Method to a Constructive One

A study by Harrison CJ (2016) demonstrated the advantages of abandoning behavioristic assessment methods that rely on incentives and punishments. It highlights the possible advantages of using three constructivist assessment tenets: step-by-step descaffolding to facilitate change towards a learning orientation, authenticity, and giving students a more active role [17].

Formative Assessment and Feedback

Kishan Prasad HL et al. (2020) concluded that by using the Objective Structured Practical Examination (OSPE) as a formative assessment tool, teaching-learning methodologies may be modified [18]. A study by Lim YS concluded that most medical students value the use of formative evaluations in teaching them the skills of self-directed learning (SDL) [19].

Wolcott MD (2018) found that when included in a curriculum for health professionals, the multiple mini interview (MMI) can be a useful tool for the thorough evaluation of professional abilities. According to this analysis, the MMI is a valid method of assessment that may be successfully integrated into the health professions curriculum to evaluate specific professional abilities [20].

A study by Hossain S et al. (2014) demonstrated that summative assessment is significantly impacted by formative assessment in several ways. The input that instructors and students receive via formative assessments is crucial to the teaching-learning process. Additionally, formative evaluations inspire pupils to study regularly and pursue in-depth knowledge. However, too many formative assessments interfere with students' ability to learn independently, which has a detrimental impact on summative assessment [21].

A study by McKenzie S. (2017) proved that checklists, together with timely feedback help learners recognize their mistakes [22]. Gonzalo JD (2014) concluded that it is important to support clinical educators in integrating tools for reflection and feedback into their bedside instruction [23]. A study by Choi S. (2020) supported the advantages of process-oriented feedback over feedback that is outcome-oriented. Process-oriented feedback aims to teach students new methods for achieving a standard instead of just telling them what is right or wrong [24].

A study by Kim EJ (2019) demonstrated that, compared to negative feedback, positive feedback resulted in higher levels of self-efficacy and stronger positive feelings. Positive reinforcement and conduct that promotes change in moderation should be a part of performance reviews, as this combination will improve both academic and emotional results [25]. Uhm S et al. (2018) concluded that medical students can learn and reflect on important information by utilizing feedback-based communication skills assessments, which may result in better communication skills [26]. Pelgrim EAM et al. (2012) proved that feedback's essence, delivery method, and integration into trainees' education are crucial [27].

Hurdles in the Implementation of Feedback

A study by Bok HJG et al. (2013) demonstrated that it was difficult to incorporate concurrent formative feedback and input for summative choices. Supervisors said that time restrictions prevented high-quality feedback from being provided, while students were reluctant to request assessments with feedback because they thought all workplace-based assessments (WBAs) were summative. The narrative formative input was deemed unhelpful by the trainees. The biggest obstacles in the upcoming years will be developing a clinical setting that is inherently supportive of feedback, such as by streamlining paperwork (e.g., by developing mobile-friendly evaluation tools) and providing supervisors and students with feedback training [28].

A study by Bates J. (2013) emphasized the need to get past the idea that assessment and feedback are just a collection of procedures and abilities and realize that, in order to be effective, these processes and abilities must be integrated into interactions and learning environments that are helpful. When assessment and feedback are longitudinal and integrated into routine patient care, they become productive. This way of thinking is especially crucial for students to embrace constructive criticism and cultivate introspective practices [29].

Harrison CJ et al. (2013) found that it seemed that better achievers used feedback more for positive reinforcement than for diagnostic data. It has been discovered that rather than trying to alter their conduct, trainees, and students were looking for comments to boost their confidence. After an exam, we must create feedback in a way that will most effectively involve the students who require the greatest assistance [30]. Feller K and Berendonk C (2020) found that feedback from supervising physicians and allied healthcare professionals (AHPs) has a compounding impact, provides insight into the performance from many angles, and helps paint a broader picture. Within the framework of workplace-based evaluation, interprofessional feedback seems to serve as a means of mutual learning [31].

Computer or Online-Based Formative Test With Automated Feedback

A study by Mitra NK et al. (2015) found that in a multidisciplinary integrated module of the third-year MBBS program, there was a favorable correlation between how well one performed in the summative assessment and the online computer-based formative assessment with automated feedback. It was determined that any rise in the usage of computer-based formative assessments with automated feedback will result in a marginal improvement in the student's summative assessment score since the learning process will be improved [32].

Bijol V (2015) demonstrated that online quizzes that provide relevant feedback, known as formative self-assessments, can assist students in evaluating their knowledge and pinpointing areas of weakness. This enables timely interventions that effectively support student learning. It was discovered that students who chose to take quizzes performed well on the final test in every category [33]. Palmer E and Devitt P. (2014) proved that the internet is a useful and well-acknowledged tool that may encourage student-centered learning and offer prompt formative feedback. Nevertheless, creating high-quality content takes time [34]. A study by Kühbeck F (2019) demonstrated that online tests that provide formative feedback enable students to more accurately measure their academic achievement and knowledge base [35]. A study by Ode GE, 2019 proved that it is possible to implement the instant feedback program using an electronic platform, and it provides replicable construct validity [36].

Video Feedback

Karn BS (2019) demonstrated that the clinical performance assessment (CPA) of medical education found that the video feedback was more useful than the analytical checklist score. Performance competence scores were greater in the experimental group that got video feedback [37]. Dohms MC (2020) proved that real-world video feedback (VF) appears to be significantly linked to an increase in one's perception of one's own empathy [38].

E-Learning Platforms for Formative Assessments

A study by Ismail MAA (2019) proved that there is potential for Kahoot! platform to help with formative evaluation in medical education. A popular free formative assessment tool in education is Kahoot!, an interactive platform for game-based learning. With Kahoot!, educators may design four distinct game-based learning experiences: surveys, jumbles, quizzes, and debates where participants compete with one another. Therefore, Kahoot! should be integrated into the educational endeavors of the health profession, especially for formative evaluation [39]. A study by Jafri L et al. (2020) found that it is prudent to promote the implementation of virtual learning environments (VLEs) in the context of the WBA program. Real-time data collection for formative assessments is now feasible because of the development of VLE and related software systems [40]. Denison A. et al. (2016) found that, when compared to the conventional, paper-based data-gathering method, the usage of tablet devices in OSCE exams is linked to better examiner comments for use as feedback [41].

The audience response system did not have a beneficial long-term effect on evaluation findings, according to research by Schmidt T. et al. (2020). The primary benefits of audience response systems would be more student motivation and the creation of an engaging learning environment than an increase in assessment scores [42].

Workplace-Based Assessment (WBA)/Mini-Clinical Evaluation Exercise (Mini-CEX)/Direct Observation of Procedural Skills (DOPS)

The study by Nair BR et al. (2015) showed that both assessors and learners said the WBA process was beneficial and offered excellent chances for performance improvement [43]. Lefroy J. (2015) researched to directly compare WBA feedback in the undergraduate medical program with and without the use of marks. 78% of middle-stage medical students expressed a desire for marks, and it was shown that marks can be beneficial when they are connected to formative evaluation [44]. Gaunt A. (2017) concluded that the degree of trainees' ambiguity over WBA's goal greatly influenced how they used it. Many trainees thought of WBA as a way to evaluate their learning, and some weren't sure what WBA was there for [45]. A study by Liang Y and Noble LM (2020) indicated that participants, in particular instructors, were not completely aware of the mini-CEX's formative aim [46]. The findings of the study by Berendonk C (2018) put a question mark on the validity of mini-CEX domain scores for formative purposes [47]. Rogausch et al. (2015) found that narrative feedback is more appropriate and seems to have more informative value than quantitative mini-CEX ratings [48]. In contrast, a study by Suhoyo Y et al. showed that, regarding the mini-CEX's usefulness and its effect on professional growth and learning, both experts and students agreed to a strong agreement [49]. Liao KC (2013) concluded that before assessors may be trained, faculty development is necessary to carry out a successful mini-CEX evaluation program [50]. The findings of the 2013 study by Tokode OM et al. about the educational aspect of mini-CEX show that participants reported learning new clinical skills, correcting incorrect clinical competencies, and increasing their knowledge via the mini-CEX assessment. The low acceptance of mini-CEX by some learners may have been caused by the high-pressure hospital setting with overlapping clinical responsibilities, the challenge of organizing the assessment, and the variable availability of time as well as the motivation of consultants to deliver quality feedback [51]. Bansal M et al. (2019) concluded that due to its ability to assess candidates at the "does" level, DOPS is an effective tool [52].

Discussion

Application of Different Constructivist Tools

Various constructivist instruments can be utilized to evaluate students' learning, performance, and advancement [53]. Some of these tools are concept maps, portfolios, and rubrics.

Concept Maps

Concept mapping allows learners to organize important ideas spatially rather than in a sequential or semantically ordered manner. It can also include iconography and visual aids. Concept maps are a useful tool for formative assessment because they allow students to create various visual representations of what they have learned [54].

Portfolios

A student's portfolio is a deliberate compilation of their own work. It is an ongoing log of the writing abilities of the pupils throughout time. Additionally, it serves as a live example for pupils to see what they have accomplished or not accomplished [55].

Rubrics

Rubrics are evaluation scales that are especially useful for assessments of tasks or performance. A rubric is a document that lists the requirements for an assignment and uses that information to clarify what is expected of it. One tool for grading student work is a rubric. Rubrics let students reflect on and assess themselves [56]. A collection of scoring instructions or descriptive scoring techniques is called a rubric. The purpose of the rubric should be to serve as a tool for formative evaluation to encourage critical thinking and enhance constructivist teaching strategies. Rubrics are a useful tool for formative assessment because they promote clear learning intentions and give instructors and students a foundation for goal setting, feedback, and peer assistance [57]. 

Increasing Student Involvement in the Design and Implementation of the Formative Assessment

Learners learn most effectively when they are actively involved in the process, driven to assess their knowledge against predetermined benchmarks, and provided with timely, focused help to meet their learning requirements. In general, this point of view supports a process of assessment that centers on the active participation of students and uses formative test results to set particular knowledge goals for both students and instructors [58].

Self-Regulatory Learning

Students taking ownership of their own learning and engaging in fruitful formative assessments may both benefit from self-regulatory learning (SRL). Through ongoing, deliberate interactions between teachers and students that were performance- and learning-directed, formative assessment helped students develop their capacity for self-regulation. Theorists concur that when learners develop the ability to adapt and self-directed learning traits necessary for greater involvement with the process of learning and subsequently good performance, SRL is associated with improved academic achievements and motivation [59]. 

Peer Review or Peer Feedback

One way that students can provide comments on each other's work is through peer evaluation or peer review. Peer review plays a significant role in fostering a culture of more active learning in the classroom. In both academic and professional contexts, peer evaluation may be employed as a tactic to increase students' interest in their own learning. The cooperative nature of peer evaluation is related to both the larger objectives of lifelong learning and professional partnership. Peer assessments in either large or small groups can serve as the basis for written or verbal peer feedback [60]. The process of conducting an efficient peer assessment is viewed as more complex than just introducing a suitable assessment tool, despite the benefits and theoretical support for peer assessment. Participants and facilitators alike acknowledge their reluctance to engage with these tools [61].

Online Formative Assessment

It has been discovered that the formative evaluation conducted online fosters student engagement and the growth of learners. To support and modify their self-regulated learning, online formative assessment also allows students to evaluate themselves and get feedback [62]. Student involvement and passion for learning were aided by e-assessments. The outcomes showed how important e-assessments were to the process of teaching and learning [63]. Additionally, it has been discovered that formative evaluation using computer-administered multiple-choice questions positively impacts student activation. Online formative assessment can take many different forms (e.g., practice tests, one-minute papers, tasks involving the clearest or muddiest points, different group projects in the classroom, etc.) [64].

Online Computer-Based Formative Test with Automated Feedback

The benefit of objective marking by predetermined scoring criteria is provided by computer-based assessment systems, which assess the results seamlessly and without regard to the subjects or assessment scenarios [65]. The benefits of computer-based assessment (CBA) in formative assessments are mostly associated with the speed and timing of computer-generated (detailed) feedback as well as the test's question selection flexibility. Higher learning outcomes may result from the ability to provide pupils with timely feedback while they are completing the exam. Electronic feedback in courses delivered online has been found to improve student learning [66].

*Use of Computerized Web-Based *Objective Structured Clinical Examination (*OSCE) Evaluation System*

Digital recordings, films, and computer files can be made of student performances. It was discovered that using electronic software made it easier to analyze the whole set of data, saving a significant amount of time. Comparably, the use of an electronic system greatly reduced the amount of time required for the results analysis, freeing up a greater amount of time for data comprehension, improved curriculum creation, and advancements in clinical teaching. The online OSCE deployment demonstrated acceptability, affordability, and viability [67]. This technique makes it easier for both the assessor and examinee to provide feedback, making it a potentially helpful tool for skill evaluation and instruction [68].

Use of Narrative Feedback Rather Than Numerical Scores in Mini-CEX

Some recent research and recommendations concluded that numerical mini-CEX scores might be removed from the forms because they don't seem to provide much information. When it comes to informing learners about changes in practice, narrative feedback works better than 'checkboxes'. It is important to look for ways to either increase the WBA ratings' informative usefulness or avoid using them altogether in favor of narrative comments alone. Directly observing student-patient interactions is valuable because it provides rich narrative feedback that sparks important conversations among learners and trainers.

A model framework of assessment for competency-based medical education that will be relevant in the Indian context (Table 3).

**Table 3:** A model framework of assessment for competency-based medical education that will be relevant in the Indian context

Formative feedback by online assessments/e-learning platforms
(i) For feedback delivery, digital mediums should be used, as they can lead to a more participatory feedback process, enhanced comprehension and higher-order thinking abilities, more genuine, supportive, and personal contact, and more detailed, tailored feedback. (ii) The use of video feedback may foster an environment of participatory assessment, build a meaningful relationship with our students, and encourage a mindset of development and attention to detail.
Virtual learning environment (VLE) for WBA
(i) Curriculum communications, assessment and progress data, and lesson plans may all be provided through the VLE (smartphones, tablets, computers, etc.). (ii) By providing immediate feedback, a VLE may generally improve formative assessment.
Peer Feedback
(i) Peer feedback enhances self-reflection and offers prompt remarks on students' work. (ii) Students also get the chance to think about what they have done and how it could be boosted when they review one another's work; (iii.) Peer review can be difficult, but, when teachers set clear expectations, students can participate in constructive peer review.
Peer‑Assisted Learning (PAL)
(i) An effective PAL technique may support both peer and self-assessment.
Embedded assessments
(i) By incorporating targeted activities that let students show off their present knowledge and abilities without needing to pause for an official exam, we can integrate assessments into their lessons.

Formative Feedback by Online Assessments/E-Learning Platforms

Assessment feedback may change as a result of digital input, especially in online learning settings. Teachers can record video or audio footage with transcripts so that students can comprehend the transcribed comments and hear the instructor's tone, in addition to providing thorough textual material entered straight into a student's digital document. A more participatory feedback process, enhanced comprehension, and higher-order thinking abilities, more genuine, supportive, and personal contact, and more detailed, tailored feedback are all advantages of receiving feedback through the digital medium. By using video feedback, you may foster an environment of participatory assessment, build a meaningful relationship with your students, and encourage a mindset of development and attention to detail [69]. 

Virtual Learning Environment (VLE) for WBA

An online platform for instructional material access is called a virtual learning environment, or VLE. Computers or smartphones can be used for this. Curriculum communications, assessment and progress data, and lesson plans may all be provided through the VLE. This makes them easily accessible to trainers and/or students, who may then utilize them to customize the learning process. It is possible to provide real-time verbal or video interaction in virtual learning environments. This will enable instruction in real-time. This could also involve some on-screen engagement, like a virtual whiteboard or screen sharing, for instance. Students will find significant value in the immediate feedback that a VLE provides following the observation stage. With this, they may use it to assume more responsibility for their own learning. By providing immediate feedback, a VLE may generally improve formative assessment [70]. 

Peer Feedback

Peer feedback enhances self-reflection and offers prompt remarks on students' work. Feedback may frequently be completed more rapidly when students evaluate one another's work than when the instructor reviews each student's work separately. Students also get the chance to think about what they have done, how it could be boosted, and whether it fulfills assignment requirements when they review one another's work. Peer review can be difficult since friendships and culture might influence it. However, when teachers set clear expectations and ask engaging questions, students can participate in constructive and beneficial peer review [71]. 

Peer‑Assisted Learning (PAL)

Activities related to peer-assisted learning (PAL) involve individuals from comparable social groups who are not certified instructors assisting one another in the learning process. PAL is widely acknowledged and used as an instructional strategy in health professional courses. It involves a socialization process and frequently involves younger and senior students serving as mentees and mentors, respectively. PAL exercises offer a structure that allows students to hone and improve their teaching and learning abilities. Students learn from and alongside one another through the utilization of common resources and the contributions of their diverse experiences. An effective learning technique may support both peer and self-evaluation [72]. 

Embedded Assessments

Assignments, exercises, or activities that are completed during training but are utilized to gather assessment information on a specific learning objective are known as embedded assessments. By incorporating targeted activities that let students show off their present knowledge and abilities without needing to pause for an official exam, teachers integrate evaluation into their lessons. The student’s work can be assessed by the instructor and/or assessors; a rubric is commonly used in this process [73].

Limitations of the study

With respect to the methodology used, there is a chance that not every accessible study will be identified in a systematic review. Despite using a strict methodology, it's possible that some research was overlooked. Additionally, research works published in other languages may have been overlooked, as only English-language publications were considered. This also applies to research in conference proceedings, book chapters, or gray literature.

We planned to use The Grading of Recommendations Assessment, Development, and Evaluation (GRADE) approach to evaluate the quality of the evidence gathered through this systematic review; however, we were unable to assess the quality of the evidence for the recommendations. The included studies' heterogeneity made it impossible to apply GRADE to assess the research's quality.

Using the GRADE approach to score the included research proved difficult, especially in cases where assessments were impacted by various circumstances in the setting of undergraduate competency-based medical education. A conscious choice was made to label these recommendations as "not rated" in response to this.

Please note that an a priori protocol was prepared before undertaking the systematic review, and it was published in the Journal of Education and Health Promotion [74].

## Conclusions

It has been noted that summative assessments are significantly impacted by formative assessments in several ways. However, too many formative assessments interfere with students' ability to learn independently, which has a detrimental impact on summative assessment. Compared to negative feedback, positive feedback results in higher levels of self-efficacy and stronger positive feelings. When assessment and feedback are longitudinal and integrated into routine patient care, they become productive. It has been discovered that rather than trying to alter their conduct, trainees, and students were looking for feedback to boost their confidence. Feedback from allied health care professionals (AHPs) has a compounding impact, provides insight into performance from many angles, and helps paint a whole picture. Any rise in the usage of computer-based formative assessments with automated feedback will result in an improvement in the student's summative assessment score since the learning process will be improved. It is possible to implement the instant feedback program using an electronic platform, and it provides replicable construct validity. It is prudent to promote the implementation of virtual learning environments (VLEs) in the context of the WBA program. Real-time data collection for formative assessments is now feasible because of the development of VLE and related software systems. The WBA approach, like all other formative assessment techniques, functions best when it is integrated into the workplace, offers targeted feedback, and is implemented timely.

The participants, in particular instructors, were not completely aware of the mini-CEX's formative aim. A question mark has been put on the validity of mini-CEX domain scores for formative purposes. This systematic literature review highlighted that formative feedback via online assessments and e-learning platforms, virtual learning environments (VLE) for WBA, peer feedback, and peer-assisted learning (PAL) are all necessary components of a model framework of assessment for competency-based medical education that will be applicable in the Indian setting. Therefore, this study proposes the following recommendations: *Formative feedback by online assessments and video feedback: *For feedback delivery, digital mediums should be used. The use of video feedback may facilitate an environment of participatory assessment.* Virtual learning environment (VLE) for WBA: *Curriculum communications, assessment and progress data, and lesson plans may all be provided through the VLE, as it generally improves formative assessment. *Peer Feedback and Peer‑Assisted Learning (PAL): *Through peer feedback, students get the chance to think about what they have done and how it could be boosted. An effective learning PAL technique may support both peer and self-assessment. ​​​​​​​*Embedded assessments: *An important tool for the improvement of learning and teaching may be embedded assessment tasks in the framework of instruction. For embedded assessment, we advise developing a toolbox.
